# Band versus Polaron: Charge Transport in Antimony Chalcogenides

**DOI:** 10.1021/acsenergylett.2c01464

**Published:** 2022-08-11

**Authors:** Xinwei Wang, Alex M. Ganose, Seán R. Kavanagh, Aron Walsh

**Affiliations:** † Department of Materials, Imperial College London, Exhibition Road, London SW7 2AZ, U.K.; ‡ Thomas Young Centre and Department of Chemistry, University College London, 20 Gordon Street, London WC1H 0AJ, U.K.

## Abstract

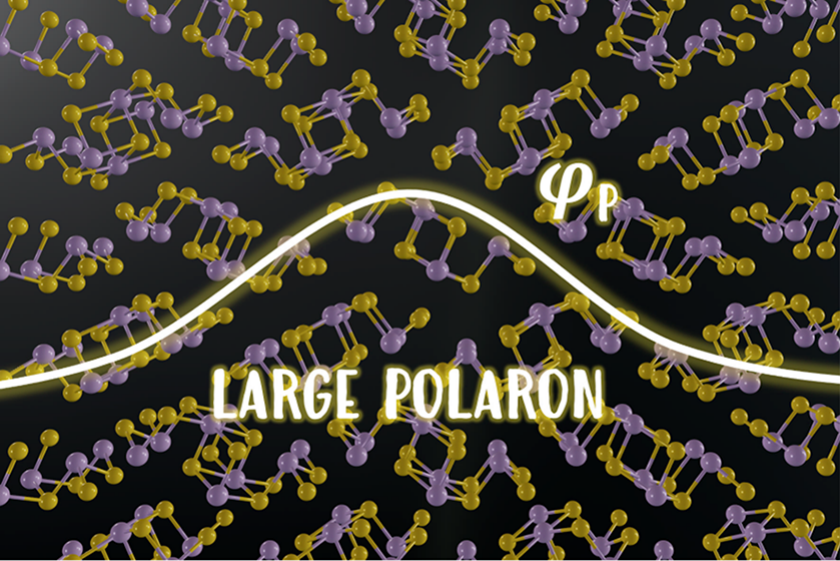

Antimony sulfide (Sb_2_S_3_) and selenide (Sb_2_Se_3_) are emerging earth-abundant absorbers for photovoltaic applications. Solar cell performance depends strongly on charge-carrier transport properties, but these remain poorly understood in Sb_2_X_3_ (X = S, Se). Here we report band-like transport in Sb_2_X_3_, determined by investigating the electron–lattice interaction and theoretical limits of carrier mobility using first-principles density functional theory and Boltzmann transport calculations. We demonstrate that transport in Sb_2_X_3_ is governed by large polarons with moderate Fröhlich coupling constants (α ≈ 2), large polaron radii (extending over several unit cells), and high carrier mobility (an isotropic average of >10 cm^2^ V^–1^ s^–1^ for both electrons and holes). The room-temperature mobility is intrinsically limited by scattering from polar phonon modes and is further reduced in highly defective samples. Our study confirms that the performance of Sb_2_X_3_ solar cells is not limited by intrinsic self-trapping.

Antimony chalcogenides (Sb_2_X_3_; X = S, Se) have emerged as promising light-absorbing materials due to their attractive electronic and optical properties, including ideal band gaps (1.1–1.8 eV) and high optical absorption coefficients (>10^5^ cm^–1^).^1−11^ They are binary compounds with earth-abundant, low-cost, and non-toxic constituents. The power conversion efficiencies in Sb_2_X_3_ solar cells have improved rapidly over the past decade, with record efficiencies reaching 7.50% and 10.12% for Sb_2_S_3_ and Sb_2_Se_3_, respectively.^12,13^ Nevertheless, efficiencies are still well below those seen in state-of-the-art CdTe or hybrid halide perovskite devices, which have reached above 25% under laboratory conditions.^14^

The underlying efficiency bottleneck is unclear. While the structural, electronic, and optical properties of Sb_2_X_3_ have been widely investigated, the charge-carrier dynamics, which critically affect conversion efficiencies, remain controversial. Charge-carrier transport in Sb_2_X_3_ has been reported in several studies,^15−19^ but there are several fundamental questions that remain unanswered. The first is whether the nature of carrier transport is band-like or thermally activated hopping. Yang et al.^15^ studied the charge-carrier dynamics in Sb_2_S_3_ and ascribed the observed 0.6 eV Stokes shift to self-trapped excitons, suggesting hopping transport. In contrast, Liu et al.^20^ and Zhang et al.^17^ argued against self-trapping in Sb_2_Se_3_ due to the saturation of fast signal decay with increasing carrier density. Considering that it is challenging for direct measurements to distinguish whether the photoexcited carriers are intrinsically self-trapped or trapped at defect sites,^21^ a systematic theoretical study on the carrier transport in Sb_2_X_3_ is necessary. The second issue is about the resulting charge-carrier mobility. Measured mobilities in Sb_2_X_3_ show a large variation,^2,19,22−26^ in part due to different synthesis and characterization methods. As such, the intrinsic limits to mobility in Sb_2_X_3_ are unclear, and the scattering physics underlying carrier transport is not yet understood.

In this work, we studied the tendency for polaron trapping and its effect on charge-carrier transport in Sb_2_X_3_ by first-principles density functional theory (DFT) and Boltzmann transport calculations. The electron–lattice interaction in Sb_2_X_3_ was explored through the Fröhlich polaron coupling constant and Schultz polaron radius. Modeling of electron and hole polarons in Sb_2_X_3_ indicates the intrinsic formation of large polarons, in contrast to recent suggestions of small polarons (i.e., self-trapped carriers).^15,16^ The prediction of large polaron formation is further reinforced by the results of carrier transport calculations. The isotropically averaged mobilities are larger than 10 cm^2^ V^–1^ s^–1^ at room temperature and decrease with increasing temperature for both electrons and holes, further confirming the band-like transport in Sb_2_X_3_. We find the intrinsic mobility is limited by scattering from polar optical phonons at low and moderate defect concentrations, while at high charged defect concentrations (>10^18^ cm^–3^) impurity scattering dominates. We expect our results will enable the design of Sb_2_X_3_ devices with improved efficiencies.

Sb_2_X_3_ crystallize in the orthorhombic *Pnma* space group and are comprised of strongly bonded quasi-one-dimensional (1D) [Sb_4_X_6_]_*n*_ ribbons oriented along the [100] direction (Figure 1). Ribbon formation is driven by the Sb lone pair, with ribbons stacked together by weak interactions.^11^ According to our previous optimization using the HSE06 hybrid functional and D3 dispersion correction,^11^ the calculated lattice parameters are 3.80/3.95 Å, 11.20/11.55 Å, and 11.39/11.93 Å for Sb_2_S_3_/Sb_2_Se_3_ along the *a*, *b*, and *c* axes, respectively. Sb_2_X_3_ are indirect band gap semiconductors with calculated indirect/direct band gaps of 1.79/1.95 eV and 1.42/1.48 eV, respectively, which are in reasonable agreement with previous experimental^1−5,27−29^ and theoretical studies.^6−9,30^ The electronic band structures are shown in Figure S1 in the Supporting Information. It has been widely suggested that efficient transport can only happen along the ribbons, based on the understanding that Sb_2_X_3_ are 1D semiconductors.^31−35^ However, neither the structural dimensionality nor the electronic dimensionality of Sb_2_X_3_ is 1D.^11,36^

**Figure 1:**
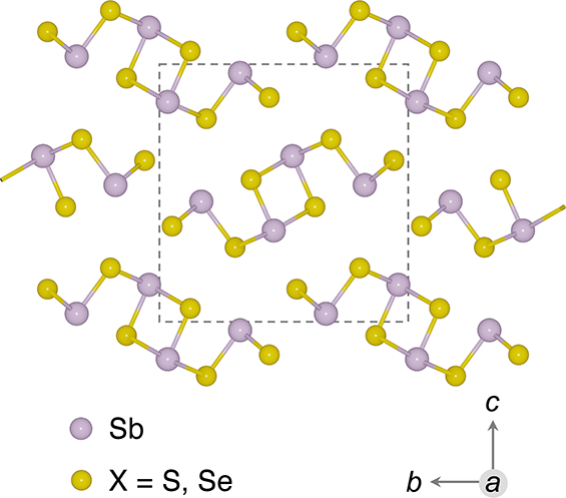
Ground-state crystal structure (*Pnma* space group) of Sb_2_X_3_. The conventional unit cell is represented by a rectangle.

Charge carriers in crystals are formally described as quasi-particles due to their interaction with the extended structure. In polar semiconductors, the charge carriers and the surrounding lattice deformation form a so-called polaron,^37^ which determines the nature of carrier transport. Polarons can be classified into two types based on the strength of electron–phonon coupling. Stronger coupling leads to larger local lattice distortion, which provides the driving force for small polarons to form. Thus, for a small polaron, the lattice deformation is usually confined to one unit cell, and a carrier’s motion is typically incoherent, with thermally activated hops which lead to low mobility (≪1 cm^2^ V^–1^ s^–1^). By contrast, the lattice deformation in a large polaron is usually moderate and spreads over multiple unit cells, resulting in a larger mobility (>1 cm^2^ V^–1^ s^–1^). In polar crystals, the electron–phonon interaction is usually dominated by the coupling of charge carriers to the longitudinal optical phonons, which can be described within the Fröhlich model.^38^

We first evaluate the Fröhlich interaction on the basis of the coupling constant α. The calculated α (shown in Table 1) shows an isotropically averaged value of ∼2 for both Sb_2_S_3_ and Sb_2_Se_3_, which falls in the intermediate electron–phonon coupling regime (defined as 0.5 ≲ α ≲ 6).^39^ The magnitudes of α along the [100] and [010] directions are quite close (Δα = 1.2–1.4 and 0.3–0.4 for electrons and holes, respectively), suggesting similar electron–phonon interaction strengths along these two directions. We further estimate the size of polarons in Sb_2_X_3_ by the Schultz polaron radius (*r*_f_).^40^ The large values of electron and hole polaron radii (which extend over multiple structural units) indicate that the polarons are delocalized in both Sb_2_S_3_ and Sb_2_Se_3_. The details of parameters used and the procedure for averaging α can be found in section S2 of the Supporting Information.

**Table 1:** Calculated Fröhlich Parameter (α) and Schultz Polaron Radius (*r*_f_) for Electrons (*e*^–^) and Holes (*h*^+^) in Sb_2_S_3_ and Sb_2_Se_3_ at *T* = 300 K

		α		*r*_f_ (Å)	
material		*e*^–^	*h*^+^	*e*^–^	*h*^+^
Sb_2_S_3_	avg	1.6	2.0	45.5	40.4
	*x*	1.0	1.8	57.3	43.7
	*y*	2.4	2.1	36.9	40.3
	*z*	5.7	2.5	23.7	36.4

Sb_2_Se_3_	avg	1.3	2.1	40.5	31.9
	*x*	0.8	2.0	50.9	32.4
	*y*	2.0	1.6	32.8	36.1
	*z*	5.8	3.8	18.8	23.5

For an alternative assessment, we performed direct first-principles DFT calculations to model charge carriers in Sb_2_X_3_. There are two challenges for reliable polaron modeling. The first is the self-interaction error^41^ arising from the approximate form of the exchange-correlation functional, which causes electrons to spuriously delocalize.^42,43^ This is typically resolved by employing a hybrid functional^44−46^ that incorporates a certain amount of exact Fock exchange or by a Hubbard correction (DFT+U).^47,48^ Second, the formation of localized polarons is dependent on the initial geometries and wavefunctions. Different methods have been proposed to break the crystal symmetry and promote the formation of localized states. Among them, the bond distortion method and electron attractor method have proved reliable across a range of structures and chemistries.^21,43,49−53^ The former involves introducing local perturbations in a supercell in a region where the polaron is expected to localize, while the latter uses a temporarily substituted atom to attract an electron or a hole, which is then removed and the structure re-relaxes. In this work, all polaron calculations were performed using the HSE06 hybrid exchange-correlation functional. We attempted to localize electron and hole polarons by adding or removing an electron from a Sb_2_X_3_ supercell using both of these distortion methods. The full computational details and workflow are provided in section S4 in the Supporting Information (Figure S3). No energy-lowering distortions were found in any case. The electrons and holes always preferred to delocalize rather than localize in both Sb_2_S_3_ and Sb_2_Se_3_ (see Figures S4 and S5), indicating again that small polarons are unlikely to form intrinsically by self-trapping. This is also supported by recent experimental evidence that the trap states in Sb_2_Se_3_ are saturated by moderate-density photocarriers.^20^

As self-trapping could originate from either self-trapped carriers (i.e., small polarons) or self-trapped excitons, we next consider the possibility of forming self-trapped excitons. First, the large dielectric constants (∼100) and small effective masses (∼0.1) in Sb_2_X_3_^11^ suggest that the Coulomb interaction is strongly screened and a large exciton radius is favored. The small experimental exciton binding energies (0.01–0.05 eV for Sb_2_S_3_ and 0.04 eV for Sb_2_Se_3_)^31,54^ further indicate weak electron–hole interactions in Sb_2_X_3_. Additionally, experimental measurements of the imaginary part of the frequency-dependent complex photoconductivity in Sb_2_Se_3_ do not reveal any negative components^55^ that can be a signal of exciton formation. Consequently, we conclude that self-trapped excitons in pristine Sb_2_X_3_ are unlikely.

To further understand the nature of transport in Sb_2_X_3_, the first-principles carrier mobility^57^ was calculated. Both *n*-type and *p*-type doping were investigated, with calculations including scattering from ionized impurities (IMP), acoustic phonons (ADP), and polar optical phonons (POP). Piezoelectric scattering was not considered due to the centrosymmetric crystal structure. The isotropically averaged mobilities are reasonably high at room temperature (*T* = 300 K) for both electrons (∼40 cm^2^ V^–1^ s^–1^) and holes (∼15 cm^2^ V^–1^ s^–1^), at low and moderate defect concentrations (<1 × 10^18^ cm^–3^), indicating band-like transport (Figure 2a). The hole mobilities are a little lower than the electron mobilities in both Sb_2_S_3_ and Sb_2_Se_3_, suggesting that *n*-type doping could be beneficial for carrier collection in photovoltaic devices. This is in contrast to experimental measurements that have indicated higher mobility for *p*-type Sb_2_Se_3_;^26^ however, this may be related to the doping asymmetry in these materials. The intrinsic mobility is limited by Fröhlich-type polar optical phonon scattering, suggesting that large polarons are responsible for the transport behavior (Figure 2b). We note that large deformation potentials have been suggested as the origin of self-trapping in the bismuth double perovskites.^58^ However, in Sb_2_X_3_, acoustic deformation potential scattering is weak (due to small deformation potentials <6 eV), similar to that seen in the hybrid halide perovskites,^59,60^ indicating self-trapping is unlikely to occur via direct coupling with acoustic vibrations.

**Figure 2:**
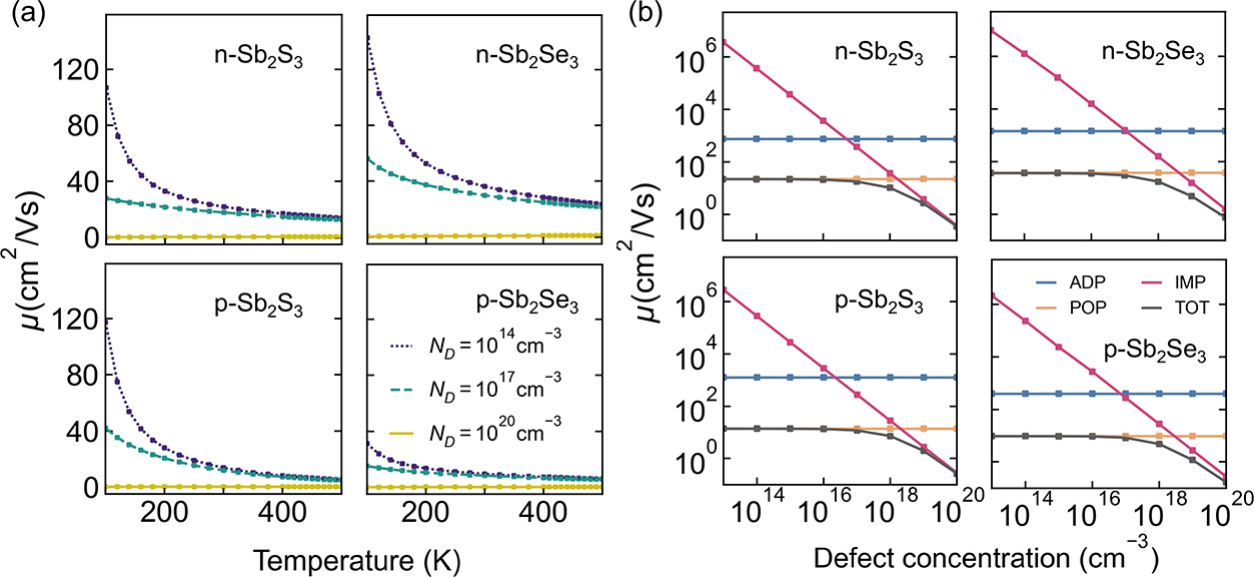
(a) Calculated average mobilities of electrons and holes in Sb_2_S_3_ and Sb_2_Se_3_ as a function of temperature with different defect concentrations. (b) Calculated total and component mobilities as a function of bulk defect concentration at 300 K. ADP, acoustic deformation potential; POP, polar optical phonon; IMP, ionized impurity; *N*_D_, defect concentration.

The scattering from ionized impurities increases with the defect concentration. At concentrations around 10^18^ cm^–3^, IMP and POP scattering are of roughly the same strength and cause the mobility to be reduced by a factor of 0.5 (Figure 2b). At higher defect concentrations, transport is entirely dominated by ionized impurities. Our results indicate that careful control of defect concentrations is essential for preventing degradation of device efficiencies. This agrees well with previous experimental reports that the defect density is crucial to the carrier transport in Sb_2_X_3_, whereby bulk defect densities above 10^15^ cm^–3^ led to significant degradation in conversion efficiency.^61−63^ Furthermore, considering that most experimental mobility measurements in Sb_2_X_3_ were obtained from thin films, where grain boundary (GB) scattering will further lower the mobility, we also tested the inclusion of mean free path scattering. According to our results (Figure S2 and Table S3), the mobilities in Sb_2_X_3_ are not significantly affected by GB scattering, even with grain sizes down to 10 nm—much smaller than the domain sizes typically seen in experiments.^64−67^ Accordingly, our results suggest that GB scattering is unlikely to be a dominant source of scattering in Sb_2_X_3_ thin films, in agreement with previous studies.^68^

The anisotropy of mobility was also considered. As shown in Table 2 and Figure 3, our calculated mobilities are in reasonable agreement with the range of measured values. For electron transport, there is considerable anisotropy, with the [100] direction showing roughly 6 times the mobility of the [010] direction and over 30 times the mobility of the [001] direction in both Sb_2_S_3_ and Sb_2_Se_3_. For holes in Sb_2_S_3_, there is a high mobility in the (001) plane, where the transport is roughly isotropic, approximately twice that in the [001] direction. For holes in Sb_2_Se_3_, the picture is slightly altered, with the highest mobility seen along [010], roughly 2 times the mobility along [100] and 8 times the mobility along [001]. The anisotropy in mobility follows the anisotropy in the calculated effective masses and the Fermi-surface dimensionality.^11^ Thus, as the electron mobilities are higher and more anisotropic than the hole mobilities, control of the grain orientation is necessary to achieve more efficient electronic transport in devices, which can be realized by strategies such as seed screening^69^ and quasi-epitaxial growth.^70^ Despite the anisotropic behavior, even at moderate defect concentrations, the electron and hole mobilities are still reasonably large (>10 cm^2^ V^–1^ s^–1^) in at least two directions. The common description of Sb_2_X_3_ as a 1D semiconductor^71,72^ oversimplifies the nature of transport. Accordingly, it may be possible to obtain high-mobility thin films, even when the grains are not fully aligned along the direction of the quasi-1D ribbons.

**Table 2:** Calculated Mobilities of Electrons (μ_*e*_) and Holes (μ_*h*_) in Sb_2_X_3_ at 300 K under Different Defect Concentrations (*N*_D_), with Experimental Values for Comparison^a^

			calcd (cm^2^ V^–1^ s^–1^)			
material			*N*_D_ = 10^14^ cm^–3^	*N*_D_ = 10^17^ cm^–3^	*N*_D_ = 10^20^ cm^–3^	exptl (cm^2^ V^–1^ s^–1^)
Sb_2_S_3_	μ_*e*_	*x*	53.90	44.72	0.96	
		*y*	9.60	7.13	0.07	
		*z*	1.88	1.35	0.01	
		avg	21.79	17.73	0.35	
		*a*_r_	28.67	33.13	96.00	

	μ_*h*_	*x*	18.58	15.90	0.38	
		*y*	13.53	11.33	0.19	
		*z*	9.34	8.35	0.22	
		avg	13.82	11.86	0.26	6.4–12.8,^2^ 32.2–54.0^25^
		*a*_r_	1.99	1.90	2.00	

Sb_2_Se_3_	μ_*e*_	*x*	89.97	76.38	1.96	
		*y*	16.74	11.65	0.11	
		*z*	1.94	1.41	0.01	
		avg	36.22	29.81	0.70	15^26^
		*a*_r_	46.38	54.17	196.00	

	μ_*h*_	*x*	9.50	8.38	0.17	2.59^56^
		*y*	16.95	14.63	0.25	1.17^56^
		*z*	2.22	1.95	0.06	0.69^56^
		avg	9.55	8.32	0.16	5.1,^22^ 3.7–21.88,^23^ 45^26^
		*a*_r_	7.64	7.50	4.17	

a The anisotropy ratio (*a*_r_) is defined as the ratio of maximum to minimum mobility.

**Figure 3:**
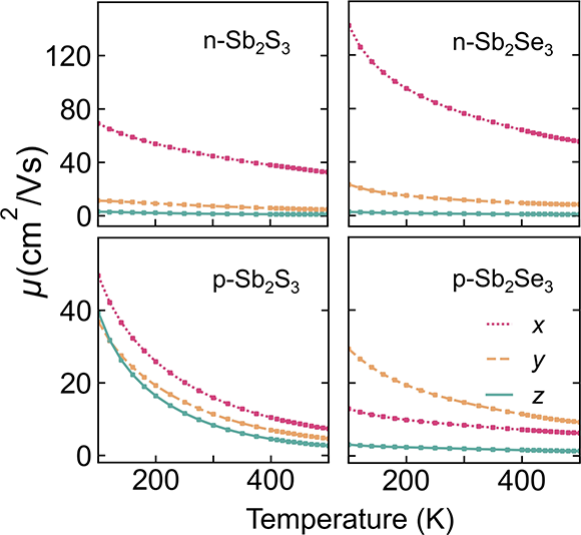
Anisotropic net carrier mobilities, including all scattering mechanisms in Sb_2_S_3_ and Sb_2_Se_3_, as a function of temperature, with a bulk defect concentration of 10^17^ cm^–3^.

Conclusively, our results show no theoretical evidence for carrier self-trapping in Sb_2_X_3_. While self-trapping has been proposed on the basis of several experimental observations,^15,73^ the formation of large polaron carriers do not contradict these observations: (i) a Stokes shift of 0.6 eV and broad photoluminescence (PL); (ii) picosecond carrier decay kinetics; (iii) absence of photoexcited carrier density saturation (up to 10^20^ cm^–3^); and (iv) polarized light emission in Sb_2_X_3_ single crystals. First, a large Stokes shift and broad PL are found in many chalcogenide semiconductors, especially those with deep defect levels, such as Sb_2_X_3_. Second, the time scale for carrier decay due to self-trapping is typically sub-picosecond or several picoseconds, while a time scale of tens of picoseconds is found in transient absorption (TA) measurements of Sb_2_X_3_.^15,73^ We note that the interpretation of TA signals and kinetics in indirect band gap semiconductors is still evolving. Third, the TA signal persists to high carrier densities (excitation power), which could also be explained by photoinduced absorption or a large trap density ⩾10^20^ cm^–3^.^17^ Finally, polarized light emission is found in many semiconductors and is connected to the underlying crystal and defect structures. Therefore, we find no evidence that directly supports intrinsic carrier self-trapping in these materials.

In summary, we investigated the nature of charge carriers in Sb_2_X_3_ semiconductors. Our results strongly suggest that self-trapping (i.e., the formation of small polarons) is unlikely to occur and that, instead, charge transport involves large polarons with moderate mobility. In particular, we found (i) intermediate Fröhlich coupling constants (∼2); (ii) large Schultz polaron radii (∼40 Å); (iii) the absence of electron or hole polaron formation in DFT calculations using the bond distortion and electron attractor methods; and (iv) carrier mobilities >10 cm^2^ V^–1^ s^–1^ at room temperature for both electrons and holes (in agreement with recent experiments). We conclude that there is no theoretical evidence for small polaron formation in pristine Sb_2_X_3_ and self-trapping is unlikely to be the origin of the low open-circuit voltages in Sb_2_X_3_ devices, as reported in previous studies.^15,16^ Accordingly, the low photovoltages may not be a bulk property of these materials and could be surmountable with improved fabrication and processing conditions to engineer the defect and interfacial properties of devices.

## Methods

The Fröhlich polaron properties were solved using the open-source package PolaronMobility.^74^ The first-principles carrier scattering rates and resulting mobilities were calculated using AMSET.^57^ The materials parameters used for these predictions are provided in Tables S1, S2, S4–S6. The crystal structure was plotted using Blender(75) and Beautiful Atoms.^76^

All of the underlying electronic structure calculations were performed based on Kohn–Sham DFT^77,78^ as implemented in the Vienna Ab initio Simulation Package (VASP).^79^ The projector-augmented wave (PAW) method^80^ was employed with a plane-wave energy cutoff of 400 eV. All calculations were carried out using the Heyd–Scuseria–Ernzerhof hybrid functional (HSE06)^81,82^ with the D3 dispersion correction,^83^ which has been proved to be able to well describe the structural and electronic properties in Sb_2_X_3_.^11^ The atomic positions were optimized until the Hellman–Feynman forces on each atom were below 0.0005 eV Å^–1^ for unit cells and 0.01 eV Å^–1^ for 3×1×1 supercells. The energy convergence criterion was set to 10^–6^ eV. Γ-centered *k*-point meshes were set to 7×2×2 and 2×2×2 for geometry optimization with primitive unit cells and supercells, respectively. For uniform band structure calculations, which were used as inputs for AMSET, a denser *k*-point mesh of 19×10×10 was used, which is consistent with our previous calculations of carrier effective masses.^11^ Detailed settings and convergence data are presented in section S6 in the Supporting Information (Figure S6 and Tables S4–S6).

## Notes

Data produced during this work has been uploaded to the NOMAD Repository and is freely available at DOI: 10.17172/NOMAD/2022.08.11-2.

## Supporting Information Available

Electronic band structures; formulas and input data of Fröhlich polaron coupling constant and Schultz polaron radius; effect of grain boundary scattering; workflow of localizing a polaron; and mobility parameters, including Figures S1–S6 and Tables S1–S6 (PDF)

## Abbreviations

GB: grain boundary
DFT: density functional theory
1D: one-dimensional
ADP: acoustic phonon
IMP: ionized impurity
POP: polar optical phonon
PL: photoluminescence
TA: transient absorption
