# Supplementary material for: Band versus Polaron: Charge Transport in Antimony Chalcogenides
Source: ACS Energy Lett. 2022 Aug 11;7(9):2954–60. doi: 10.1021/acsenergylett.2c01464 (PMC9469203; doi:10.1021/acsenergylett.2c01464)
Supplement: Supplementary file 1 — nz2c01464_si_001.pdf [file nz2c01464_si_001.pdf]

Supporting Information
Band Versus Polaron: Charge Transport in Antimony Chalcogenides

Xinwei Wang,[†] Alex M. Ganose,[†] Seán R. Kavanagh,[†,‡] and Aron Walsh[*,†]

† *Department of Materials, Imperial College London, Exhibition Road, London SW7 2AZ, UK*

‡ *Thomas Young Centre and Department of Chemistry, University College London, 20 Gordon Street, London WC1H 0AJ, UK*

E-mail: a.walsh@imperial.ac.uk

S1. Electronic band structures

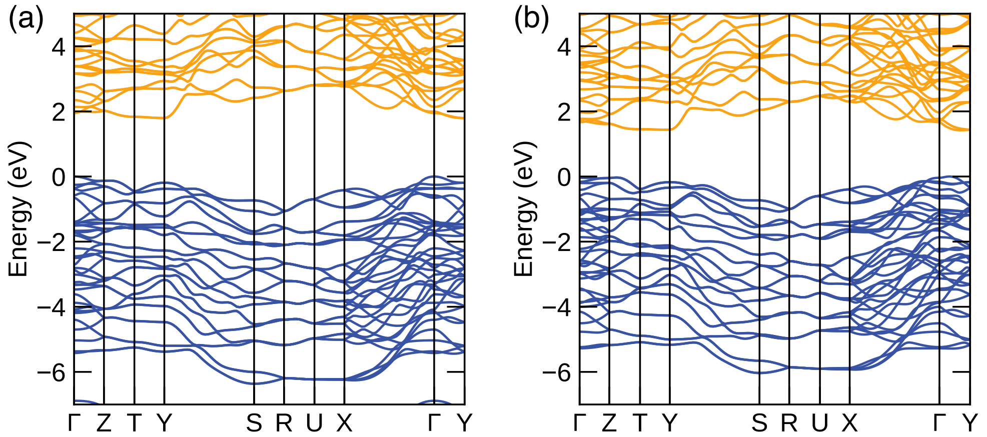

Figure S1: Electronic band structures of (a) Sb_2S_3 and (b) Sb_2Se_3.

S2. Fröhlich polaron coupling constant and Schultz polaron radius

Table S1: Parameters used to calculate Fröhlich polaron coupling constant α. The effective phonon frequency (ω) is in THz

Material		ε_∞	ε_0	ω	m^*	
					e	h
Sb_2S_3	avg	10.26	68.76	3.49	0.40	0.64
	x				0.16	0.47
	y				0.92	0.65
	z				5	0.97
Sb_2Se_3	avg	13.52	76.27	2.57	0.35	0.90
	x				0.14	0.85
	y				0.81	0.55
	z				7	3

The long-range electron-longitudinal optical phonon coupling can be expressed by the dimensionless Fröhlich polaron coupling constant α [1]

$$\alpha = \frac{e^2}{\hbar}\left(\frac{1}{\varepsilon_\infty} - \frac{1}{\varepsilon_0}\right)\sqrt{\frac{m^*}{2\hbar\omega}}, \tag{1}$$

where ε_∞ and ε_0 are the high-frequency and static dielectric constants, respectively, m^* is the effective mass and ω is the effective phonon frequency. The effective mass and effective frequency were calculated using the AMSET package.[2] The isotropic α was obtained using the harmonic mean of the effective masses and the arithmetic average of the dielectric constants. The anisotropic α was calculated using the anisotropic (direction-dependent) effective masses, consistent with previous work.[3]

Table S2: Parameters used to calculate Schultz polaron radius (r_f, Å)

Material		α		v		w		m^*_{P}	
		e^-	h^+	e^-	h^+	e^-	h^+	e^-	h^+
Sb_2S_3	avg	1.6	2.0	12.70	12.98	11.16	10.99	0.52	0.89
	x	1.0	1.8	12.34	12.79	11.39	11.11	0.19	0.62
	y	2.4	2.1	13.25	12.99	10.82	10.98	1.38	0.91
	z	5.7	2.5	16.07	13.30	9.32	10.79	14.88	1.47
Sb_2Se_3	avg	1.3	2.1	16.72	17.29	15.32	14.96	0.42	1.20
	x	0.8	2.0	16.40	17.24	15.53	14.99	0.16	1.12
	y	2.0	1.6	17.21	16.95	15.01	15.17	1.06	0.69
	z	5.8	3.8	20.68	18.68	13.05	14.12	17.59	5.25

Schultz polaron radius is defined as[4]

$$r_f = \sqrt{\frac{3}{2\mu v}}, \tag{2}$$

$$\mu = \frac{v^2 - w^2}{v^2}, \tag{3}$$

where v and w are Feynman-model variational parameters which specify the polaron state. They are solved variationally by the Feyman polaron model using Fröhlich polaron coupling constant α as an input. μ is the reduced effective mass.

S3. Effect of grain boundary scattering

Table S3: Calculated mobilities of electrons (μ_e) and holes (μ_h) in Sb_2X_3 at 300 K with and without grain boundary scattering. The anisotropy ratio (a_r) is defined as the ratio of maximum to minimum mobility

Material			Calculated mobility ($cm^2\,V^{-1}\,s^{-1}$)		
			Mean free path (nm)		
			-	100	10
Sb_2S_3	μ_e	x	44.72	43.98	38.57
		y	7.13	7.07	6.55
		z	1.35	1.34	1.25
		avg	17.73	17.46	15.45
		a_r	33.13	32.82	30.86
	μ_h	x	15.90	15.77	14.71
		y	11.33	11.25	10.58
		z	8.35	8.29	7.82
		avg	11.86	11.77	11.04
		a_r	1.90	1.90	1.88
Sb_2Se_3	μ_e	x	76.38	74.56	62.14
		y	11.65	11.46	10.07
		z	1.41	1.39	1.23
		avg	29.81	29.13	24.48
		a_r	54.17	53.64	50.52
	μ_h	x	8.38	8.29	7.57
		y	14.63	14.41	12.78
		z	1.95	1.93	1.81
		avg	8.32	8.21	7.38
		a_r	7.50	7.47	7.06

The effect of grain boundary scattering on the mobility in Sb_2X_3 was evaluated by incorporating an average grain size using the AMSET package.[2] The grain boundary scattering lifetime is set to v_g/L, where v_g is the group velocity and L is the mean free path. In this work, the mean free path of 10 and 100 nm were tested. The carrier concentration and defect concentration were assumed to be $10^{13}\,cm^{-3}$ and $10^{17}\,cm^{-3}$, respectively.

According to our results (Table S3 and Fig. S2), at temperatures between 100 and 500 K, the total mobility is not limited by the grain boundary scattering. The anisotropic values at room temperature are shown in Table S3. After considering the grain boundary scattering,

the values of anisotropy ratio change slightly and the most favourable directions for carrier transport remain the same for both Sb_2S_3 and Sb_2Se_3.

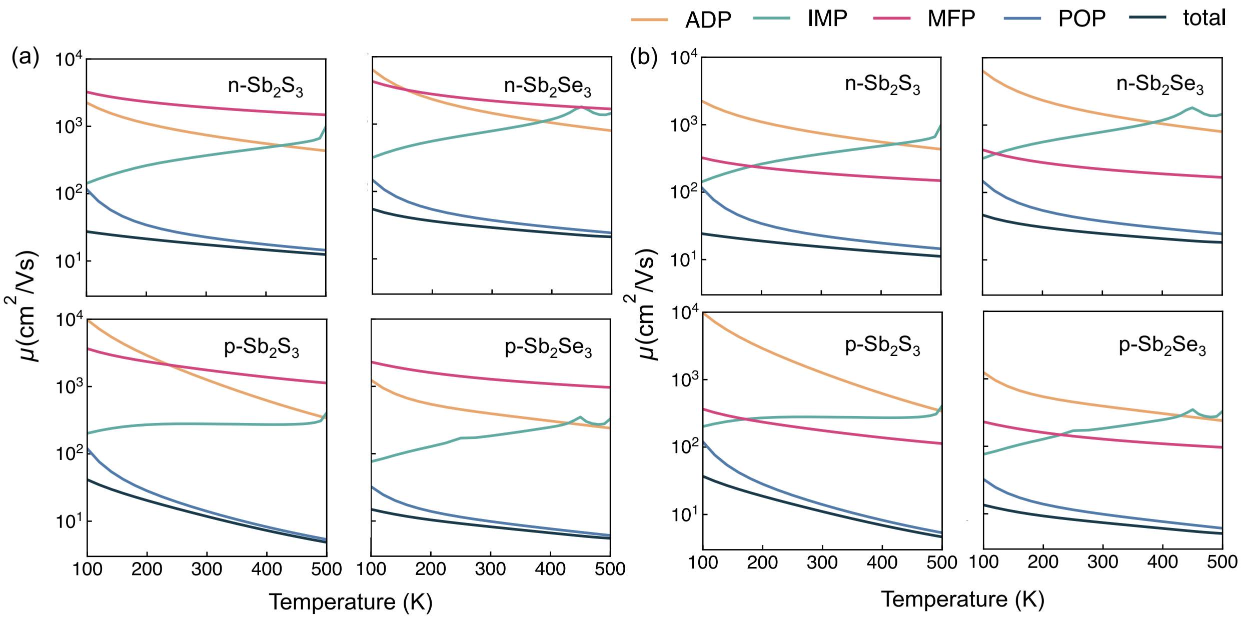

Figure S2: Calculated component and total mobilities with mean free path of (a) 100 and (b) 10 nm as a function of temperature.

S4. Workflow of localising a polaron in Sb_2X_3

We attempted to localise an electron or a hole in Sb_2S_3 and Sb_2Se_3 by the bond distortion method and electron attractor method (Fig. S3). A 3×1×1 supercell (with the dimension of 11.40×11.20×11.39 $Å^3$ and 11.85×11.55×11.93 $Å^3$ for Sb_2S_3 and Sb_2Se_3, respectively) was constructed, which is sufficient to model small polarons.[5–7] In each system, one electron per supercell was added or removed to introduce an electron or a hole.

We first applied the bond distortion method to introduce distortions around one designated atom (Sb for adding an electron and S/Se for adding a hole) for each non-equivalent Sb and S/Se. These are implemented by the ShakeNBreak package.[8,9] Different distortions of 20%, 30% and 40% with both compression and stretching were considered. However, after structural optimisation, all lowest-energy structures relaxed to perfect configurations.

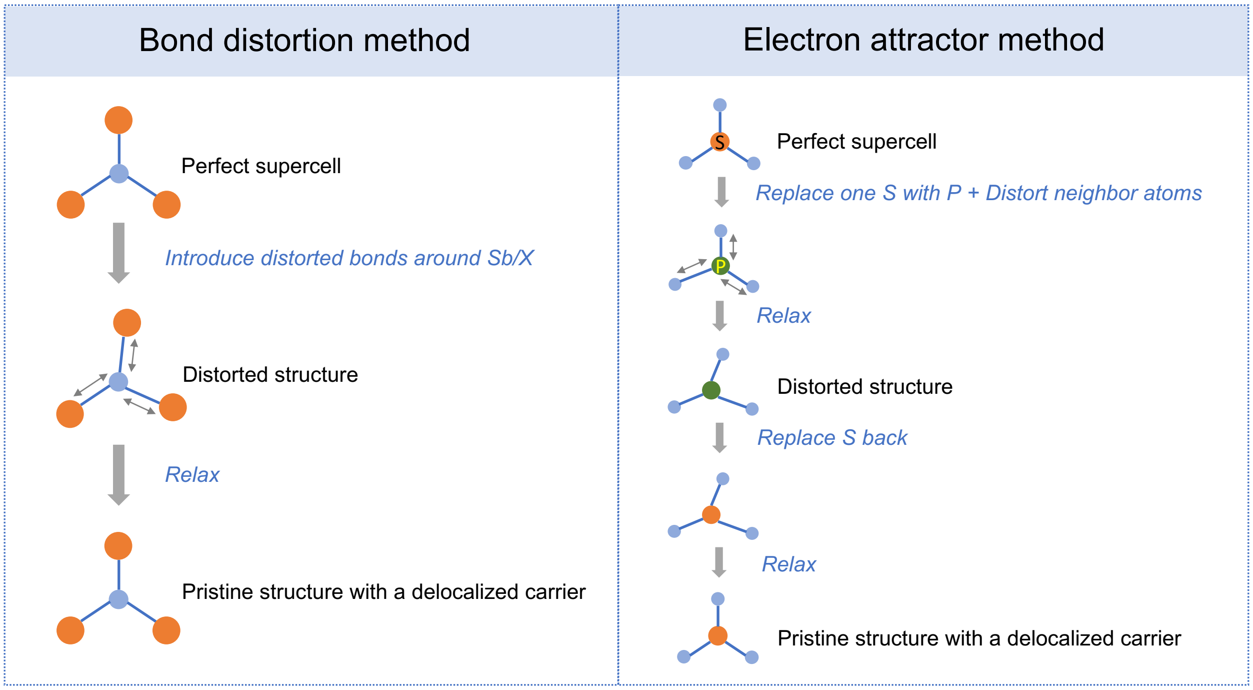

Figure S3: The workflow of bond distortion method and electron attractor method.

We further combined the bond distortion method with the electron attractor method to confirm the formation of hole polarons in Sb_2S_3. The electron attractor method refers to attracting electrons or holes to a particular atomic site by replacing one certain atom. Phosphorous has stronger attraction to holes than sulfur as it contains fewer protons and has a lower electronegativity. Here, we used one P to replace one S in a supercell, introduced some local distortions around the P atom and add small random displacements to all atoms to break the symmetry in the initial structures. Three non-equivalent S sites were considered, and a range of distortions of both compression and stretching between 0% and 60% with 10% as an interval were tested. The number of electrons were kept the same as the neutral replaced system, suggesting one extra hole in Sb_2S_3. The structures with the substituted atom and local distortions were fully relaxed. Finally, for each non-equivalent S case, we used the lowest-energy structures among different distortions, replaced back the S atom and relaxed the configuration again. Nevertheless, all structures went back to perfect configurations, indicating that the localised polarons are unlikely to form.

Nevertheless, we note that using a k-point mesh of 1×1×1 to do geometry relaxation could lead to localised solution in some cases (Fig. S4a). While after converging it with denser k-point mesh of 2×2×2, we finally get delocalised polarons (Fig. S4b).

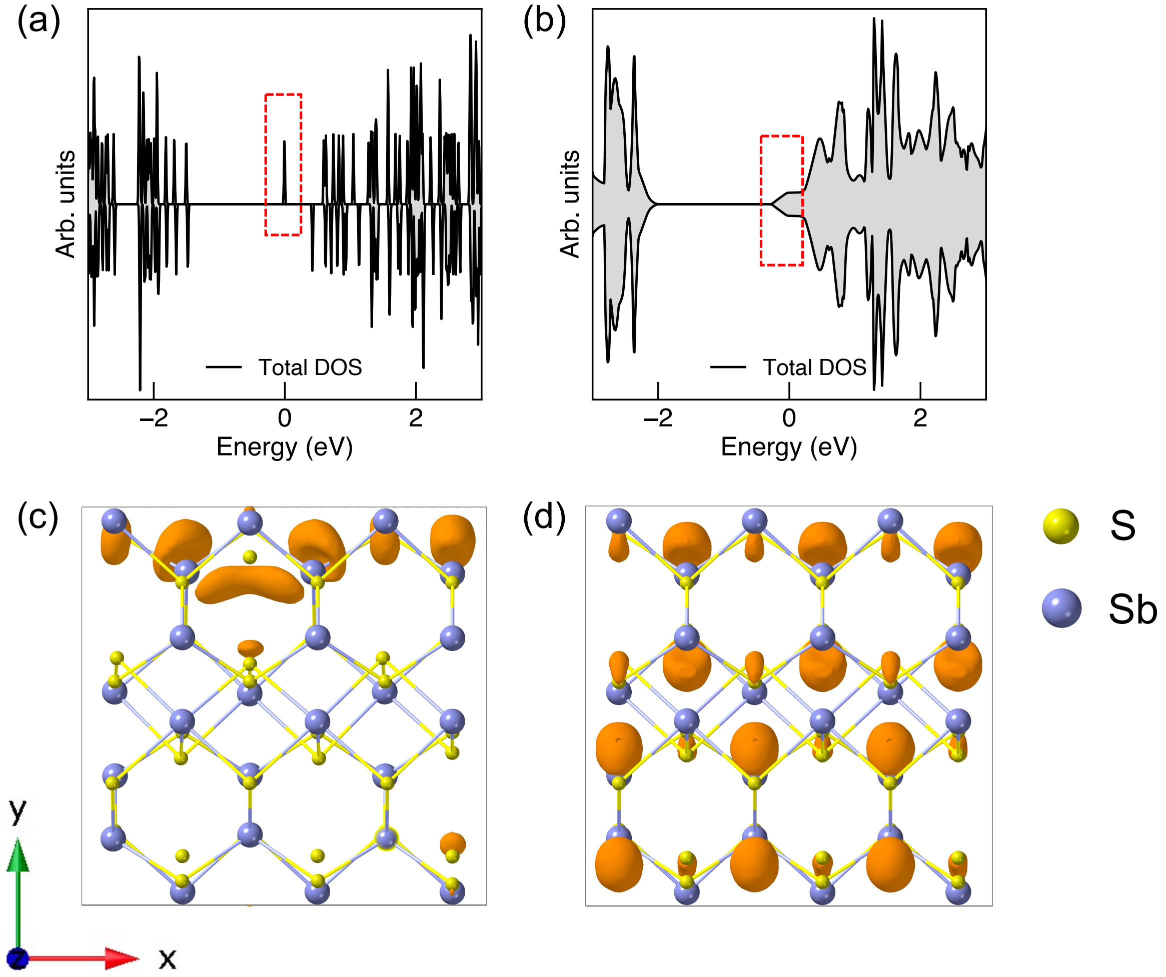

Figure S4: (a-b) Density of states and (c-d) partial charge densities of conduction band maximum for electron polarons in Sb_2S_3 using a k-point mesh of (a) and (c) 1×1×1, (b) and (d) 2×2×2 to relax structures. The partial charge densities were plotted by specifying the energy range of the conduction band maxima, which are represented by red dashed rectangles. The isosurface value for partial charge densities is set to 0.01 $e/\text{Å}^3$.

S5. Partial charge densities of electron and hole polarons

Partial charge densities of the valence band maximum (VBM) for hole polarons and conduction band maximum (CBM) for electron polarons in Sb_2X_3 are shown in Fig. S5, which are delocalised in all cases.

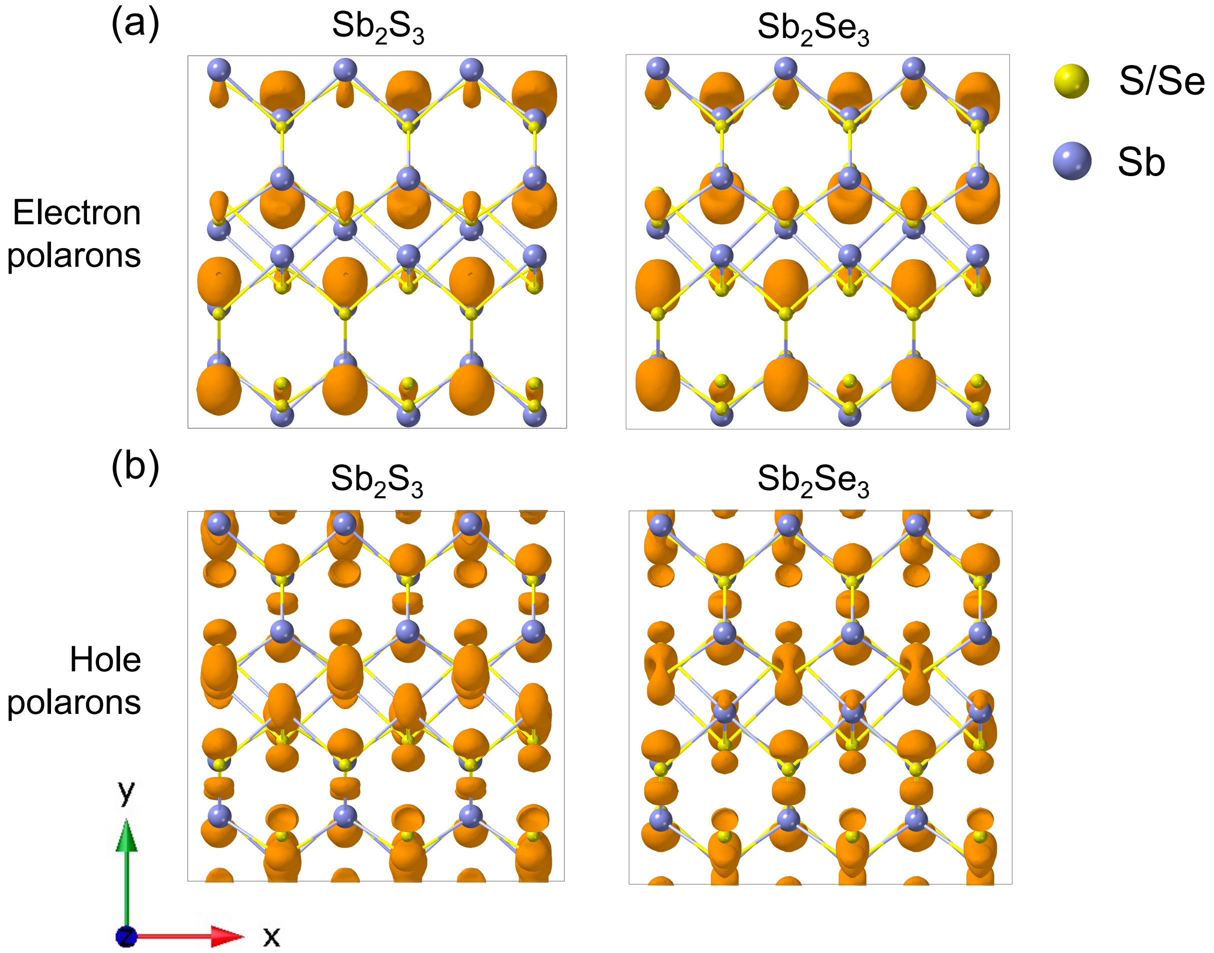

Figure S5: Partial charge densities of (a) conduction band maximum for electron polarons and (b) valence band maximum for hole polarons in Sb_2S_3 and Sb_2Se_3. The isosurface values for partial charge densities are set to 0.01 and 0.005 $e/\text{Å}^3$ for electron and hole polarons, respectively.

S6. Parameters used to calculate mobilities in Sb_2X_3

The k-point meshes used to calculate transport properties were tested (shown in Fig. S6) and a k-point mesh of 169×57×57 is used for all calculations. The carrier concentration was set to $10^{13}\,cm^{-3}$ according to previous experimental results in Sb_2X_3.[10–16] The calculated effective phonon frequency is 3.49 for Sb_2S_3 and 2.57 for Sb_2Se_3. The calculated deformation potentials, elastic constants and dielectric constants are shown in Table S4, S5 and S6, respectively.

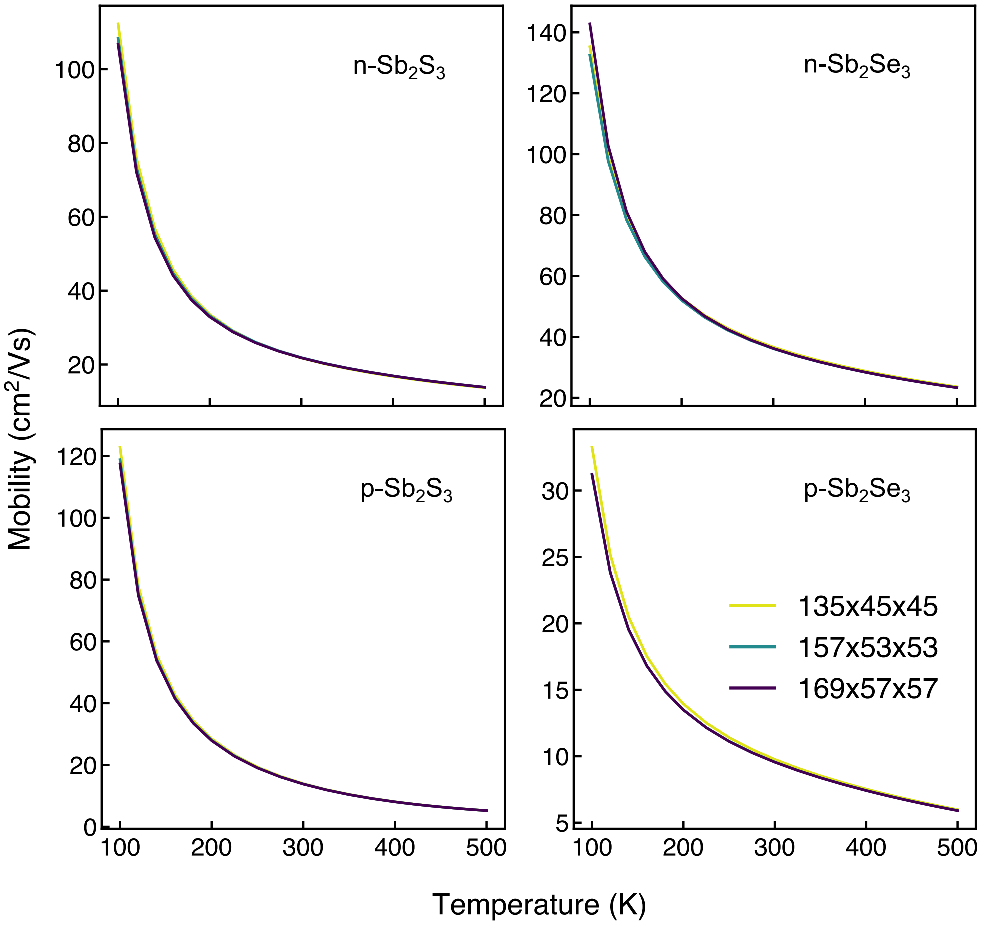

Figure S6: The convergence of mobility in Sb_2X_3 under different k-point meshes. The defect concentration is set to be $10^{14}\,cm^{-3}$ and the temperature is set to be 300 K.

Table S4: Calculated deformation potentials (D, eV) for the upper valence and lower conduction bands of Sb_2S_3 and Sb_2Se_3

Material			D_{XX}	D_{YY}	D_{ZZ}
Sb_2S_3	VBM	D_{XX}	5.41	0.26	0.07
		D_{YY}	0.26	0.10	0.02
		D_{ZZ}	0.07	0.02	1.27
	CBM	D_{XX}	5.26	0.42	0.17
		D_{YY}	0.42	2.43	3.35
		D_{ZZ}	0.17	3.35	2.62
Sb_2Se_3	VBM	D_{XX}	0.53	0.16	0.05
		D_{YY}	0.16	2.86	0.03
		D_{ZZ}	0.05	0.03	2.47
	CBM	D_{XX}	3.31	0.36	0.09
		D_{YY}	0.36	0.39	0.29
		D_{ZZ}	0.09	0.29	1.38

Table S5: Calculated elastic constants (in GPa) of Sb_2S_3 and Sb_2Se_3

Material		C_{XX}	C_{YY}	C_{ZZ}	C_{XY}	C_{YZ}	C_{ZX}
Sb_2S_3	C_{XX}	93.75	28.00	18.50	0.00	0.00	0.00
	C_{YY}	28.00	57.25	15.39	0.00	0.00	0.00
	C_{ZZ}	18.50	15.39	37.69	0.00	0.00	0.00
	C_{XY}	0.00	0.00	0.00	31.68	0.00	0.00
	C_{YZ}	0.00	0.00	0.00	0.00	17.11	0.00
	C_{ZX}	0.00	0.00	0.00	0.00	0.00	8.77
Sb_2Se_3	C_{XX}	77.15	25.63	17.11	0.00	0.00	0.00
	C_{YY}	25.63	54.15	17.03	0.00	0.00	0.00
	C_{ZZ}	17.11	17.03	31.75	0.00	0.00	0.00
	C_{XY}	0.00	0.00	0.00	23.42	0.00	0.00
	C_{YZ}	0.00	0.00	0.00	0.00	18.41	0.00
	C_{ZX}	0.00	0.00	0.00	0.00	0.00	5.08

Table S6: Calculated static (ε_0) and high-frequency (ε_∞) dielectric constants of Sb_2S_3 and Sb_2Se_3

Material	ε_0			ε_∞		
	x	y	z	x	y	z
Sb_2S_3	98.94	94.21	13.14	11.55	10.97	8.25
Sb_2Se_3	85.64	128.18	15.00	15.11	14.92	10.53

References

(1) Fröhlich, H. Interaction of electrons with lattice vibrations. *Proc. Math. Phys. Eng.* **1952**, *215*, 291–298.

(2) Ganose, A. M.; Park, J.; Faghaninia, A.; Woods-Robinson, R.; Persson, K. A.; Jain, A. Efficient calculation of carrier scattering rates from first principles. *Nat. Commun.* **2021**, *12*, 1–9.

(3) Guster, B.; Melo, P.; Martin, B. A.; Brousseau-Couture, V.; de Abreu, J. C.; Miglio, A.; Giantomassi, M.; Côté, M.; Frost, J. M.; Verstraete, M. J., et al. Fröhlich polaron effective mass and localization length in cubic materials: degenerate and anisotropic electronic bands. *Phys. Rev. B* **2021**, *104*, 235123.

(4) Schultz, T. Slow electrons in polar crystals: self-energy, mass, and mobility. *Phys. Rev.* **1959**, *116*, 526.

(5) Sun, L.; Huang, X.; Wang, L.; Janotti, A. Disentangling the role of small polarons and oxygen vacancies in CeO_2. *Phys. Rev. B* **2017**, *95*, 245101.

(6) Ding, H.; Lin, H.; Sadigh, B.; Zhou, F.; Ozolins, V.; Asta, M. Computational investigation of electron small polarons in α-MoO_3. *J. Phys. Chem. C* **2014**, *118*, 15565–15572.

(7) Castleton, C. W.; Lee, A.; Kullgren, J. Benchmarking density functional theory functionals for polarons in oxides: Properties of CeO_2. *J. Phys. Chem. C* **2019**, *123*, 5164–5175.

(8) Mosquera-Lois, I.; Kavanagh, S. R. In search of hidden defects. *Matter* **2021**, *4*, 2602–2605.

(9) Mosquera-Lois, I.; Kavanagh, S. R.; Walsh, A.; Scanlon, D. O. Identifying the ground state structures of point defects in solids. *arXiv preprint arXiv:2207.09862* **2022**, (accessed on 25/07/2022).

(10) Chen, C.; Bobela, D. C.; Yang, Y.; Lu, S.; Zeng, K.; Ge, C.; Yang, B.; Gao, L.; Zhao, Y.; Beard, M. C., et al. Characterization of basic physical properties of Sb_2Se_3 and its relevance for photovoltaics. *Front. Optoelectron.* **2017**, *10*, 18–30.

(11) Liu, M.; Gong, Y.; Li, Z.; Dou, M.; Wang, F. A green and facile hydrothermal approach for the synthesis of high-quality semi-conducting Sb_2S_3 thin films. *Appl. Surf. Sci.* **2016**, *387*, 790–795.

(12) Zhou, Y.; Leng, M.; Xia, Z.; Zhong, J.; Song, H.; Liu, X.; Yang, B.; Zhang, J.; Chen, J.; Zhou, K., et al. Solution-processed antimony selenide heterojunction solar cells. *Adv. Energy Mater.* **2014**, *4*, 1301846.

(13) Yuan, C.; Zhang, L.; Liu, W.; Zhu, C. Rapid thermal process to fabricate Sb_2Se_3 thin film for solar cell application. *Sol. Energy* **2016**, *137*, 256–260.

(14) Li, J.; Huang, J.; Li, K.; Zeng, Y.; Zhang, Y.; Sun, K.; Yan, C.; Xue, C.; Chen, C.; Chen, T., et al. Defect-resolved effective majority carrier mobility in highly anisotropic antimony chalcogenide thin-film solar cells. *Sol. RRL* **2021**, *5*, 2000693.

(15) Chalapathi, U.; Poornaprakash, B.; Park, S.-H. Influence of post-deposition annealing temperature on the growth of chemically deposited Sb_2S_3 thin films. *Superlattices Microstruct.* **2020**, *141*, 106500.

(16) Black, J.; Conwell, E.; Seigle, L.; Spencer, C. Electrical and optical properties of some $M_2^{V\text{-}B}N_3^{VI\text{-}B}$ semiconductors. *J. Phys. Chem. Solids* **1957**, *2*, 240–251.
